# A three-component cognitive behavioural lifestyle program for preconceptional weight-loss in women with polycystic ovary syndrome (PCOS): a protocol for a randomized controlled trial

**DOI:** 10.1186/s12978-017-0295-4

**Published:** 2017-03-06

**Authors:** G. Jiskoot, S.H. Benneheij, A. Beerthuizen, J.E. de Niet, C. de Klerk, R. Timman, J.J. Busschbach, J.S.E Laven

**Affiliations:** 1 Division Reproductive Medicine, Department of Obstetrics and Gynaecology, Erasmus MC, PO Box 2040, 3000 CA Rotterdam, The Netherlands; 2 Department of Psychiatry, Section of Medical Psychology and Psychotherapy, Erasmus MC, PO Box 2040, 3000 CA Rotterdam, The Netherlands

**Keywords:** Polycystic ovary syndrome, PCOS, Obesity, Life style, Quality of life, Weight loss, Cognitive therapy, CBT, Text messaging, E-health

## Abstract

**Background:**

Obesity in women with polycystic ovary syndrome (PCOS) negatively affects all clinical features, and a 5 to 10% weight loss has shown promising results on reproductive, metabolic and psychological level. Incorporating a healthy diet, increasing physical activity and changing dysfunctional thought patterns in women with PCOS are key points in losing weight. The biggest challenge in weight management programs is to achieve a reasonable and sustainable weight loss. The aim of this study is to explore whether Cognitive Behavioural Therapy (CBT) by a mental health professional, working in a multidisciplinary team with a dietician and a physical therapist (a three-component intervention), is more effective for weight loss in the long term, within 12 months. We will also explore whether mobile phone applications are effective in supporting behavioural change and sustainable weight loss.

**Methods:**

The present study is a longitudinal randomized controlled trial (RCT) to study the effectiveness of a three-component 1-year cognitive-behavioural lifestyle intervention in overweight/obese women with PCOS. A total of 210 participants are randomly assigned to three groups: 1) CBT provided by the multidisciplinary team or; 2) CBT provided by the multidisciplinary team and Short Message Service (SMS) or; 3) usual care: encourage weight loss through publicly available services (control group). The primary aim of the 12-month intervention is to explore whether a three-component 1-year cognitive-behavioural lifestyle intervention is effective to decrease weight, when compared to usual care. Secondary outcomes include: the effect of the intervention on the PCOS phenotype, waist circumference, waist to hip ratio, ovulation rates, total testosterone, SHBG, free androgen index (FAI), AMH, hirsutism, acne, fasting glucose, blood pressure and all psychological parameters. Additionally, we assessed time to pregnancy, ongoing pregnancies, clinical pregnancies, miscarriages and birth weight.

All outcome variables are measured at the start of the study, and again at 3 months, 6 months, nine months and 12 months.

**Discussion:**

We expect that CBT provided by a multidisciplinary team, especially combined with SMS, is effective in developing a healthy lifestyle and achieving a long-term weight loss in women with PCOS. Losing 5– 10% body weight improves various PCOS characteristics. Consequently, we expect to show that CBT provided by a multidisciplinary team improves reproductive and metabolic outcomes, as well as quality of life, while at the same time being cost-effective.

**Trial registration:**

Registered at the Netherlands National Trial Register with number NTR2450 on August 2nd, 2010.

## Plain English summary

Obesity in women with polycystic ovary syndrome (PCOS) negatively affects all clinical features, and a 5 to 10% weight loss has shown promising results on reproductive, metabolic and psychological level. Incorporating a healthy diet, increasing physical activity and changing dysfunctional thought patterns in women with PCOS are key points in losing weight. The present study is a longitudinal randomized controlled trial (RCT) to study the effectiveness of a three-component 1-year cognitive-behavioural lifestyle intervention in overweight/obese women with PCOS. We will also explore whether mobile phone applications are effective in supporting behavioural change and sustainable weight loss. The primary aim of the 12-month intervention is to explore whether a three-component 1-year cognitive-behavioural lifestyle intervention is effective to decrease weight, when compared to usual care. We expect that CBT provided by a multidisciplinary team, especially combined with SMS, is effective in developing a healthy lifestyle and achieving a long-term weight loss in women with PCOS. Losing 5– 10% body weight will improve various PCOS characteristics. Consequently, we expect to show that CBT provided by a multidisciplinary team improves reproductive and metabolic outcomes, as well as quality of life, while at the same time being cost-effective in women with PCOS.

## Background

Polycystic ovary syndrome (PCOS) is a common endocrine disorder that affects 5–10% of women in their reproductive years [1]. According to the ESHRE/ASRM Rotterdam consensus [2], the diagnosis of PCOS requires at least 2 of the following 3 criteria: oligoovulation or anovulation (irregular or no menstrual cycle at all), clinical (hirsutism) and/or biochemical signs of hyperandrogenism (elevated free androgen index or elevated testosterone levels), polycystic ovarian morphology (on ultrasound), and the exclusion of other aetiologies that might cause hyperandrogenism. PCOS women will generally experience one or more of the following symptoms in varying degrees: hirsutism (excessive body hair growth), acne, anovulatory infertility, obesity, insulin resistance and dyslipidaemia [2, 3].

The incidence of overweight and obesity in PCOS women is between 50 and 60% [3]. The prevalence of obesity in the general population is increasing, and this might result in an even higher incidence of PCOS in the future [4, 5]. The current prevalence of overweight and obesity is significantly higher in PCOS women, especially in Caucasian women [6]. Overweight in women with PCOS negatively affects all clinical features [3, 7–9] and gaining weight aggravates also psychological aspects. Women with PCOS report a major impact on their quality of life (QoL) due to PCOS symptoms [10, 11] and experience more distress compared to women without PCOS [12]. Weight concerns in particular appear to have the largest impact on QoL, compared to other PCOS symptoms such as amenorrhea, oligomenorrhea, hirsutism and acne [13]. Obesity is also a risk factor for lower self-esteem and greater sexual dissatisfaction in women with PCOS, compared to age matched controls [14, 15]. PCOS women with amenorrhoea seem to have lower levels of self-esteem and greater fear of negative appearance compared to PCOS women with oligo-amenorrhea [16].

Whether PCOS women have a unique predisposition to obesity is not yet clear [17]. A recent study by Louwers et al, looking into the genetic predisposition for overweight or obesity, did not discover any differences in the number of risk alleles for obesity between women with PCOS and the controls [18]. Although women with PCOS generally do have a healthy diet, they seem to have a higher caloric intake and are physically less active, compared to controls without PCOS [19]. Also, women diagnosed with PCOS more often use a self-initiated inadequate diet than controls [20]. Insulin resistance might play a mediating role in the effect of obesity on metabolic and reproductive symptoms in PCOS [21]. Other authors do suggest that the high prevalence of obesity might be a result of selection and referral bias of obese PCOS women [22]. In summary, much about the link between PCOS and obesity remains unknown [1]. It is still unclear whether obesity is the cause, or an effect of the disease itself [6, 23].

There is a large number of small (one or two-component) studies demonstrating that losing 5 to 10% of initial body weight improves reproductive, metabolic and psychological features in PCOS women [24]. Moreover, it often leads to ovulation and subsequent pregnancy, [7, 25–27] as well as a reduction in miscarriage rates in PCOS women [4, 7]. Weight loss also reduces the risk of Type II Diabetes Mellitus and the incidence of the metabolic syndrome in the long term [7, 27]. Additionally, studies have indicated that decreasing intra-abdominal fat tissue in particular results in the restoration of ovulation [28], even when women remain within their World Health Organisation (WHO) weight class after weight loss [29, 30]. Recent work from Mutsaerts et al [31] and Dokras et al [32] showed different results. A mean weight loss of 4.4 kg following a two-component lifestyle treatment of 26 weeks did not result in a significant difference in live birth rates compared to a weight loss of 1.1 kg in the control group. Also, there was no difference in pregnancy and neonatal complications between groups [31]. Weight loss by the Look AHEAD protocol (16 weeks) or the use of the oral contraceptive pill shows significant improvement in both groups at physicial and mental domains related to quality of life, depression and anxiety [32]. At the ESHRE/ASRM PCOS meeting in 2010, a consensus was reached that lifestyle should be optimized before conception [23] to improve the effectiveness of fertility treatment [33, 34] and to improve (mental)health across a woman’s lifespan as well as that of her child to be [27]. However, no international evidence based protocol exists for the long-term treatment of overweight and obesity in this particular group of obese women [35].

The biggest challenge in weight management programs is to achieve a reasonable and sustainable weight loss [36, 37]. Many obesity interventions compare one-component (physical activity or nutrition intervention) with two-component (physical activity and nutrition or nutrition and counselling) interventions. Three-component (physical activity, nutrition and counselling) lifestyle interventions seems to have the biggest effect, compared to one or two components [38]. Weight loss programs in general seem to be effective in the short term [39]; however, most of the initial weight loss is regained within 1 year [36]. Long term weight-loss seems the biggest challenge for the “global obesity epidemic” according to the WHO [40]. Incorporating a healthy diet, increasing physical activity and changing dysfunctional thinking patterns in women with PCOS are key points in losing weight [41]. However, treatment adherence is often low [36] and drop-out rates are high. The longer the treatment, the higher the chance for participants to drop-out, and indeed the highest dropout rates are reported in interventions that last 24 weeks or longer [42]. This relation between the drop-out rate and the duration of therapy is particular worrying, as long lasting lifestyle changes are preferred. Patients who are likely to drop out will benefit most from adherence to a long-term lifestyle program compared to patients not at risk to drop out. Fauser et al. commented that more research is needed to optimize lifestyle interventions, maximizing weight loss and minimizing drop-out rates for PCOS women with a wish to conceive [23].

A possible solution to increase therapy adherence and reduce the drop-out rate is support by mobile phone applications. Weight loss interventions making use of internet and mobile phone applications have emerged to induce behavioural changes [43]. There is a growing body of literature on Short Message Service (SMS) and smart phone interventions for obesity treatment, indicating that tailored text messages are more effective than generic ones [44, 45]. Smart phone and mobile phone applications seem effective when embedded in an intervention program [46]. Studies have indicated that sending SMS results in weight maintenance up to 12 months after completion of cognitive behavioural treatment (CBT), [47] and supports controlling the desire to eat and promoting an active lifestyle [48]. Moreover, the use of SMS has shown to improve adherence and decrease the drop-out rates in weight loss treatments [49], which is associated with improvement in weight-related behaviours and weight outcomes [50].

Fauser et al. commented that more research is needed to optimize lifestyle interventions, maximizing weight loss and minimizing dropout for PCOS women with a wish to conceive [23].

## Methods/design

### Aim

The aim of this study is to examine whether CBT, provided by a mental health professional working in a multidisciplinary team with a dietician and a physical therapist (a three-component intervention), is effective to decrease weight, compared to usual care at the end of treatment in obese women with PCOS. Furthermore, we explore whether mobile phone applications are effective in supporting behavioural change and sustainable weight loss.

### Research hypotheses

Primary:

1. A multidisciplinary 1-year cognitive-behavioural lifestyle intervention (with or without SMS) is more effective to decrease weight in 12 months, compared to usual care.

Secondary:

2) A multidisciplinary 1-year cognitive-behavioural lifestyle intervention (with or without SMS) is effective to decrease weight by 4.0 BMI points at 12 months, compared to usual care.
3) SMS maintenance treatment in combination with a multidisciplinary 1-year cognitive-behavioural lifestyle intervention is more effective than the multidisciplinary 1-year cognitive-behavioural lifestyle intervention alone, in terms of weight loss maintenance and drop-out reduction.
4) A multidisciplinary 1-year cognitive-behavioural lifestyle intervention (with or without SMS) is effective in improving the menstrual cyclicity as well as anthropomorphometric, ultra-sonographic, endocrine and psychological parameters in women with PCOS.

### Design

**Fig. 1:**
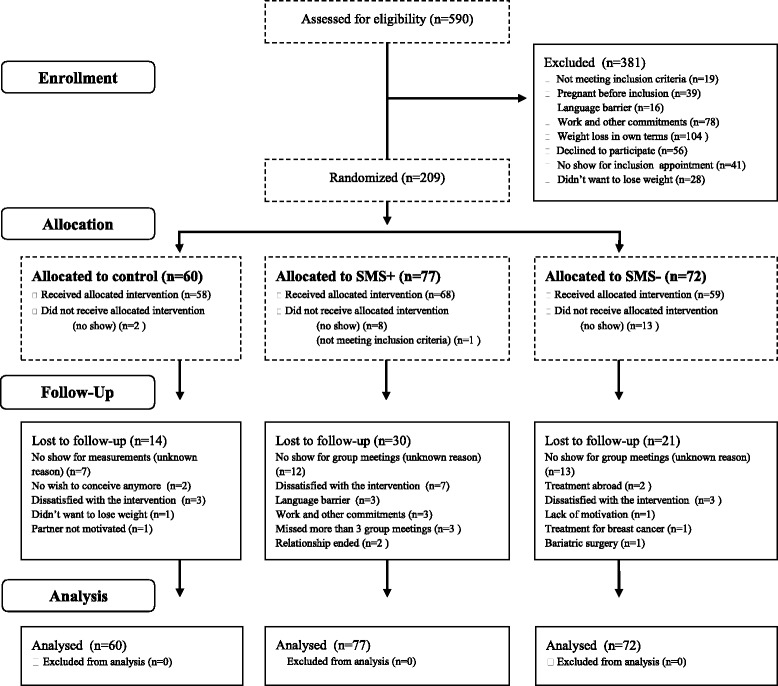
CONSORT 2010 standard RCT flow diagram

The present study is a longitudinal RCT measuring the effectiveness of a three-component multidisciplinary 1-year cognitive-behavioural lifestyle intervention in overweight/obese women with PCOS. It is a three-armed RCT comparing three groups: 1) CBT provided by a multidisciplinary team or; 2) CBT provided by a multidisciplinary team and Short Message Service (SMS) or; 3) usual care: encouraged to lose weight by publicly available services (control group), see Fig. 1.

Patients will be included in the trial at the department of Obstetrics and Gynaecology of the Erasmus MC in Rotterdam, the Netherlands.

### Participants

Women with menstrual cycle disorders are systematically screened using the same standardised protocol i.e. the so called CyclusOLigoAmenorroe (COLA) protocol. The COLA protocol includes a family and reproductive history, antropomorphometric, ultrasonographic assessments and screening. Based on this screening, women are diagnosed according the World Health Organisation (WHO) classification. Women with WHO 2 normogonadotropic normo-estrogenic anovulation are further classified using the Rotterdam consensus criteria for PCOS and non-PCOS. Patients who meet the inclusion and exclusion criteria (shown in Table 1) are eligible. At enrolment, all patients receive detailed information about the role of weight loss in PCOS and the benefits of lifestyle modification.

**Table 1:** Eligibility

Inclusion criteria:	Exclusion criteria:
✓PCOS according to Rotterdam consensus criteria [2]	✓Women with inadequate command of the Dutch language
✓Body mass index (BMI) > 25 kg/m^2^	✓Mental illness
✓Age between 18 and 38 years	✓Obesity with another somatic cause
✓Trying to conceive over 1 year	✓Ovarian tumours that lead to an androgen excess
	✓Adrenal diseases
	✓Other malformations of internal genitalia
	✓Pregnancy

The gynaecologist presents the patient information about the study using a patient information leaflet. After 2 weeks, patients are contacted by telephone in order to assess whether they are willing to participate in the study. In order to participate, patients need to sign the informed consent form and return it by regular mail. As soon as this form is received out our research office, appointments for baseline measurements are made.

### Randomisation

At the baseline, after the initial assessment, participants are randomized at a 2:1 ratio into the following groups: 1-year CBT lifestyle intervention with SMS (*n* = 78), 1-year CBT lifestyle intervention without SMS (*n* = 78), or the control group who receives usual care (*n* = 78) using a computer-generated random numbers table. A research nurse, who is not involved in the study, carries out the randomisation. Assignment is made by sequentially numbered, identical, sealed envelopes, each containing a letter designating the allocation (i.e. “A” for intervention with SMS, “B” for intervention without SMS and “C” for control,).

### Intervention

The 1-year multidisciplinary lifestyle intervention aims at: 1) changing cognitions; 2) changing dietary habits; 3) encouraging and promoting physical activity; and 4) activating social support; and consists of twenty 2.5 h group sessions. The first 1.5-h of every group session is supervised by a mental health professional and a dietician. The last hour of each session is supervised by a physical therapist. Each lifestyle intervention group consists of a maximum of 10 patients to ensure that there is sufficient individual attention for every participant. Additionally, participants receive five individual counselling sessions of 45 min with a mental health professional, five individual sessions with a physical therapist and five with a dietician.

We developed the “PCOS lifestyle textbook” for participants, which describes the activities of each group session and the homework assignments. To standardize the treatment and to facilitate the therapist’s treatment adherence, we developed a therapist manual, which includes protocols for each session. The manual also includes PowerPoint slides to present at each group session.

#### Phase 1: first 3 months (session 1 to 11)

We divided the 20 group sessions into four phases. The outline of each session is presented in Table 2. In the first phase of the program, the group sessions are held weekly. CBT techniques are used to create awareness and to restructure dysfunctional thoughts about lifestyle (food & exercise), weight (loss) and self-esteem. The Dutch food guide (DFG) is used as a guideline for healthy eating and is the main guideline during the nutritional sessions run by the dietician. Subjects receive the DFG guide for women aged 19 to 50 (Table 3) [51]. Based on the DFG, a healthy woman with a normal weight and regular exercise, may take in up to 2000 kilocalories in total, consisting of 1700 kilocalories for breakfast, lunch and dinner, and another 300 kilocalories for (healthy) snacking. The take-up of 2000 kilocalories in total is normally advised for weight maintenance. Hence, for most women in the lifestyle program this (reduced intake) will result in weight loss. During phase 1, we emphasize that participants should start making healthy, sustainable food choices and to avoid “restrained” behaviour like skipping meals and reducing food variance [52].

**Table 2:** Overview of the content of the lifestyle intervention

Session	Topic	Objectives
1	Introduction	Introduction of the therapists and the group members. Agreement on attendance, commitment, homework assignments, privacy and buddy system. Providing information about the program and target weight. Providing information about the concept of energy balance. Explaining the link between thoughts, feelings and behaviors.
2-4	Diet: Explaining the Dutch food guide and daily amounts Psychology: introduction of cognitive behavior therapy	Providing information about the Dutch Food Guide (DFG). Increasing or decreasing daily amounts based on the Dutch Food Guide (DFG) Identifying unhealthy food choices. Understanding the rationale of cognitive behavior treatment. Completing a thought record. Setting realistic goals (proactive coping).
5	Partner and social support meeting	Welcome to spouses (or family/friends) General introduction of the lifestyle program for spouses (or family/friends). How to be supportive in a helpful way. Providing information about the concept of energy balance.
6,7	Diet: Reading food nutrition labels Psychology: Biased perception and interpretation	Participants are able to read food nutrition labels. Making healthier choices based on food nutrition labels. Identifying and correcting biased perception and interpretation.
8	Dealing with infertility	Sharing emotions concerning infertility. Reframing negative thoughts concerning infertility.
9,10	Binge eating	Learning different incentive values of food. Obtaining a regular eating pattern (breakfast, lunch and dinner). Knowing the difference about hunger, appetite sensations and binge eating.
11	Partner and social support meeting	Using different preparation techniques for cooking. Asking help from spouses.
12	Binge eating	Finding alternate ways to handle binge eating Eating healthy portion sizes.
13	Diet: dealing with the holidays or special occasions Psychology: Behavioral experiment	Making a meal plan for the holidays. Explaining the rationale for behavioral experiments. Setting up behavioral experiments.
14	Mindful eating	Knowing the difference between conscious and unconscious eating. Introduction of mindfulness techniques.
15	Diet: Snacks Psychology: Relapse prevention	Learning the difference between healthy and unhealthy snacking. Identifying difficult moments and finding alternative thoughts. Reframing dysfunctional cognitions concerning relapse.
16	Diet: Eating in a restaurant Psychology: assertiveness	Eating a healthy meal in a restaurant. Performing a roll-playing game in a restaurant. Saying no in a food related situation.
17	Partner and social support meeting	Doing the healthy eating quiz. Doing a cue exposure exercise involving sweets and cookies.
18,19	Diet: Healthy pregnancy Psychology: Relapse prevention	Identifying and preventing relapse moments. Developing self-efficacy beliefs for the future. Dealing with food and appetite sensations during pregnancy.
20	Evaluation of the program Relapse prevention	Evaluation of the lifestyle program and participants progress. Providing information about long-term weight maintenance. Coping with regaining weight.

**Table 3:** Dutch Food Guide (women, age group 19–50 years)

Vegetables	200 g
Fruit	200 g (2 servings)
Bread	6 slices
Potatoes, rice, pasta or beans	200 g
Milk	450 ml
Cheese	1,5 slice
Meat	100 g
Butter and oil	30 g
Drinks	1,5 liter
(Healthy) Snacks	300 calories

The physical therapist encourages participants to use exercise as part of their daily routine, [53] according to the Global Recommendations for physical activity by the World Health Organisation, [54] and advises:

- To do 5 days of moderate physical activity for 30 min each day; and
- To do vigorous exercise 1 to 3 days a week, for at least 20 min per session; and
- To perform 8 to 10 muscle-strengthening activities involving major muscle groups twice a week.

#### Phase 2: month 3 to 6 (session 12 to 16)

In the second phase of the program, the group sessions are held once every two weeks. During this phase participants are motivated to develop a structured eating pattern to avoid over-restriction and under-restriction, like binge eating and restrained eating [55]. The frequency of face-to-face contact decreases over time, to stimulate participants to maintain healthy eating and physical activity. This is based on the principles of ‘proactive coping’ to promote self-regulation [56]. Also, behavioural skills developed during phase 1 of the intervention are reinforced [57].

#### Phase 3: month 6 to 9 (session 17 to 19)

In the third phase of the program, the group sessions are held once a month. Participants learn about relapse management and maintenance of their weight loss. By using proactive coping, participants set new goals for the next 3 months aimed at maintaining a healthy lifestyle. Individual counselling sessions are planned if needed at the request of the participant.

#### Phase 4: final 3 months (session 20)

During the final 3 months of the program, there are no scheduled group sessions. Participants can contact the multidisciplinary team if individual counselling sessions are needed.

There is an outreach policy to motivate participants to come to the measurement sessions, unless the participant indicates to withdraw from the study. Participation in the lifestyle intervention terminates if the participant misses more than 3 out of 20 group sessions. In such cases, the measurements will also stop. For obvious reasons, the intervention and the measurements will also stop when the participant is pregnant.

#### Maintenance intervention by SMS

At the 3 month point, participants are randomly assigned to SMS support or CBT without SMS support. Participants will be sending weekly self-monitored information regarding their diet, physical activity and emotions by SMS to the mental health professional for the next 9 months (Table 4).

**Table 4:** Text message plan

1) How many hours of exercise did you had in the past week? For example cycling or walking	
A) Less than 1 h	
B) 1 to 3 h	
C) 4 to 6 h	
D) 7 to 10 h	
E) More than 10 h	
2) How many days did you keep up a healthy diet in the past week?	
A) None of the 7 days	
B) 1 to 2 days	
C) 3 to 4 days	
D) 5 to 6 days	
E) All 7 days	
3) How often did you felt satisfied about yourself in the past week?	
A) Never	
B) Almost never	
C) Sometimes	
D) Often	
E) Always	
4) How often did you felt sad or unhappy in the past week?	
A) Always	
B) Often	
C) Sometimes	
D) Almost never	
E) Never	
5) What is your weight today in kilograms? For example 88,4 kg	

A semi-automated software program generates feedback in response to the incoming messages. These feedback messages provide social support, encourage positive behaviour and empower behavioural strategies. The mental health professional assesses whether the suggested feedback is applicable before sending it to the participant. In addition, participants receive two messages per week addressing eating behaviour (self-monitoring, barriers, binge eating, eating pace, emotional eating, food choices, portions, planning, preparation, stimulus control, social eating, sugar sweetened beverages) and physical activity (motivation, fun facts, sedentary behaviour). There are five types of messages, as shown in Table 5.

**Table 5:** Types of text messages

• Tips (i.e. Going grocery shopping today? Don’t go if you’re hungry!)
• Reminders (i.e. Read the flyer about the Dutch food guide again. What’s your focus this week?)
• Educational facts (i.e. Did you know that cleaning the house is also a moment of exercise? You can burn up to 140 calories in an hour!)
• Motivational messages (i.e. Are you not completely satisfied with your diet today? Tomorrow is new day, don’t give up!)
• Knowledge based (i.e. Do you know the second step in writing a thought record?)

### Control group: usual care

Just like the lifestyle intervention group, the control group visits the hospital after the initial assessment during the 4 consecutive occasions at which they are similarly assessed as the CBT lifestyle intervention group. During these 5 measurement moments they have a short, unstructured consult with their treating physician. Participants in the control group are encouraged to lose weight through publicly available services (i.e. diets, visiting a dietician, going to the gym or participating in public programs such as Weight Watchers®) and use simple strategies, including self-monitoring of their diet. The physician also mentions the risk of overweight for both mother and child, and the relation between overweight and fertility. If patients fail to achieve their target weight during the 12-month study period (see below), they can participate in the lifestyle intervention, but are not included in the trial.

At the Erasmus MC, patients diagnosed with PCOS receive ovulation induction treatment when shifted to a lower BMI category. Meaning: 1) a weight loss of 4.0 BMI points; 2) a BMI < 34.0; and 3) weight loss maintenance over 3 months. The intervention group receives ovulation induction treatment after 1) a weight loss of 4.0 BMI points; 2) a BMI < 34.0; and 3) complying with the intervention group for more than 1 year.

### Outcome measures

The primary outcome of this study is to test whether CBT provided by a mental health professional, working in a multidisciplinary team with a dietician and a physical therapist (a three-component intervention), is effective to decrease weight compared to usual care (control) at the end of the treatment. Secondary outcomes include: reproductive, drop-out, quality of life, healthy diet, physical activity, metabolic and endocrine improvements, the health of the (unborn) child. All outcome variables are measured at the start of the study, and again at 3 months, 6 months, 9 months and 12 months. All outcome measures are displayed in Table 6. Below we give more details about the collection of the secondary outcomes.

**Table 6:** Outcomes measures (T0, T1, T2, T3, T4)

Primary outcome	
Weight in kilograms	
Secondary outcomes	
(1) reproductive outcomes:	a) cycle duration and frequency
	b) spontaneous pregnancies
2) anthropomorphometric outcomes:	a) BMI
	b) waist circumference (WC)
	c) hip circumference (HC)
	d)WC/HC ratio
(3) clinical and biochemical PCOS features:	a) hair growth pattern
	- Ferriman Gallwey score
	b) blood pressure
	c) transvaginal ultrasound outcomes
	- ovarian volume
	- follicle count
	- total follicles per ovary
	d) endocrine outcomes
	- insulin resistance
	- hyperandrogenism
	- dyslipidemia
(4) psychological outcomes:	a) eating behaviour and disturbances
	- The Dutch Eating Behaviour Questionnaire (NVE)
	- Eating Disorder Examination Questionnaire (EDE-Q)
	b) quality of Life
	- Short Form 36 (SF-36)
	- Polycystic Ovary Syndrome Questionnaire (PCOSQ)
	c) depression
	- Beck Depression Inventory (BDI)
	d) self-esteem
	- Rosenberg Self Esteem Scale (RSE)
	e) fear of negative appearance
	- Fear of Negative Appearance Evaluation Scale (FNAES)
	f) satisfaction
	- Self-constructed questionnaire
(5) physical activity:	a) perceived level of daily physical activity
	- the International Physical Activity Questionnaire (IPAQ)
	b) the intervention group: submaximal bicycle test
(5) physical activity:	a) food intake
	-energy (calories)
	- protein
	- fat
	- saturated fat
	- carbohydrates
	- mono- and disaccharides
	- drinks (ml)
(7) other:	- attendance at meetings
	**-** drop-out and reasons for drop-out

#### Psychological outcomes

##### Eating behaviour and disorders

The Dutch Eating Behaviour Questionnaire (DEBQ) is a validated questionnaire; it is administered to assess restrained eating (10 items), emotional eating (13 items), and external eating (10 items). Subscales are computed as the mean of the relevant questions resulting in a score between 1 and 5, with a higher score reflecting a higher degree of the relevant eating behaviour [58]. Additionally, the Eating Disorder Examination Questionnaire (EDE-Q) is used to measure specific eating disorders. This questionnaire consists of 36 items measuring five subscales: concerns about shape, weight, and eating, in addition to restrained and binge eating. The subscale scores range between 0 and 6. A higher score indicates more severe eating psychopathology [59].

##### Quality of life

The Short Form 36 (SF-36) is a generic quality of life questionnaire that consists of 36 questions. Quality of life is divided into in eight dimensions: physical functioning, role-physical, bodily pain, general health, vitality, social functioning, role-emotional and mental health. The sum of the SF-36 item scores within each dimension is transformed into a scale ranging from 0 (poor health) to 100 (good health) [60].

The Polycystic Ovary Syndrome Questionnaire (PCOSQ) is a specific health-related quality of life (HRQoL) questionnaire for PCOS women [13]. This questionnaire consists of 26 questions and five domains: emotions, body hair, weight, infertility and menstrual problems. Each item is scored on a scale between 1 and 7, whereby 1 indicates the worst health status and 7 denotes best health status. We have translated this questionnaire into Dutch. This questionnaire has not yet been validated.

##### Depression

The Beck Depression Inventory (BDI) [61] is a validated questionnaire that measures depression and depressive symptoms according to the criteria of the DSM-IV [62]. The Dutch BDI-II [63] questionnaire consists of 21 questions for the evaluation of cognitive, affective and somatic symptoms of depression. Higher total scores indicate more severe depressive symptoms.

##### Self-esteem and body image

Self-esteem is measured by the Rosenberg Self Esteem Scale. This questionnaire consists of 10 questions and has been validated for the Dutch population [64]. The RSES measures global self-esteem. Items are scored on a 4-point scale. Scores below 21 indicate low self-esteem. The Fear of Negative Appearance Evaluation Scale (FNAES) [65] is a short questionnaire consisting of six items that measure body image, eating disorder and depression. The items are answered on a five-point Likert scale, ranging from ‘not at all’ to ‘extremely’, whereby a higher score indicates more fear of negative evaluation by others. The Dutch version has not been validated yet.

#### Dietary outcomes

Food intake is documented at home on paper, in a 3 day (including 1 weekend day) food diary to determine changes over time in food intake, meal and eating pattern, meal frequency, amount of carbohydrates, vegetables, fruit, dairy and snacks. Additionally, using a self-constructed questionnaire according to the dietary history method [66], participants are asked about:

- Weight: i.e. birth weight, highest weight, development of obesity
- Dieting: i.e. history of weight loss dieting and how much weight loss was achieved
- History of eating: i.e. how parents dealt with food while growing up
- Eating pattern: i.e. watching TV while eating, amount of take-out foods

The validity of the dietary history method during weekdays has been proven, and it is less reliable during weekends [67].

The Dutch Food Composition Database (NEVO) is used to calculate the nutritional composition of each 3 day food record. This database contains information on food products and meals that are regularly eaten by a large proportion of the Dutch population.

#### Physical activity outcomes

To gain insight into the perceived level of daily physical activity, we use the International Physical Activity Questionnaire (IPAQ) [68]. This questionnaire consists of 31 items about the frequency and duration of physical activity at work, during transportation, during household activities and during leisure time in the course of the previous week.

A continuous progressive submaximal test is used to determine the exercise intensity and the fitness progress in the intervention group. The maximum heart rate and the maximum load are determined during this test. Firstly, the resting heart rate is measured when sitting in a chair. Secondly, the heartrate is measured using a standard ramp protocol on a bicycle after a 5 minute warm-up (20 watt) on a bicycle. Thirdly, the load is increased every minute with 10, 15 or 20 watt, based on the level of the participant. During the test, the participant must maintain a speed of 60 to 80 revolutions per minute. The test is stopped when the speed is decreased by 15 revolutions per minute. The response to the submaximal test is evaluated immediately after the test by the modified Borg scale [69]. This scale ranges from 0 to 10 and provides insight into the perceived exertion level.

Prior to the submaximal test, the treating physician will examine contraindications (cardiac and/or pulmonary problems) using the Physical Activity Readiness Questionnaire (PAR-Q) [70]. In case of the slightest suspicion of cardiac and/or pulmonary problems the submaximal test is not performed.

### Sample size calculation

The original sample size calculation in 2009 was based on an anticipated effect of a difference between the groups of 0.45 in terms of Cohen’s d in the primary outcome variable (BMI), with a power (1-beta) of 0.80 and an alpha level of 0.05 (two-sided) in a 2:1 ratio. This ratio was required for analysis of the secondary outcome: the effect of SMS within the intervention group. This resulted in 156 patients to be enrolled in the intervention group and 78 patients in the control group, a total of 234. This number was registered at the Dutch Trial Registry (TC 2450). On behalf of the Grant Foundation (MRace), an interim analysis was performed in May 2014. After inclusion of 150 patients, we applied an interim power analysis to the complete cases. The control group had a reduction from 33.3 ± 6.8 kg/m^2^ to 32.6 ± 6.6 kg/m^2^, an effect of Cohen’s d = 0.10, whereas the lifestyle intervention group showed a reduction from 33.8 ± 4.8 kg/m^2^ to 31.3 ± 5.1 kg/m^2^, an effect of d = 0.52 and a difference of 0.42. For the sample size calculation, we applied the method described by Aberson [71], with a power of 0.90, a two-sided alpha of 0.025 (corrected for the interim analysis) and five repeated measures linearly decreasing. We observed an intercorrelation of about 0.90 between all measurements. Maintaining a ratio of 2:1, the required sample is 84 participants in the lifestyle intervention and 42 in the control group, a total of 126 complete cases. With an observed drop-out proportion of 40%, a total of 210 participants are needed for the study. We anticipated to have a relatively high drop-out rate, because pregnancy leads to exclusion. Note that this sample size calculation is a conservative number, as it is based on a complete case analysis of variance. The intended multilevel analysis provides more power.

### Statistical analyses

Mixed modelling will be applied for longitudinal analyses of the data by using SPSS version 21. Mixed modelling can efficiently deal with missing data and unbalanced time-points [72]. This analysis will include two levels: the patients will constitute the upper level, and their repeated measures the lower level. First, for each outcome variable a saturated model will be postulated, with the primary or/and secondary outcomes as dependent variables. The saturated models will include treatment group, time, quadratic time, logarithm of time, and all treatment-time interactions as fixed effects. The deviance statistic [73] using restricted maximum likelihood [74] will be applied to determine the covariance structure. Next, the saturated fixed part of the models will be reduced by eliminating insignificant fixed effects using Wald tests, respecting that interaction effects must be nested under their main effects [75]. The significance of the difference between the saturated models and the parsimonious final models will be determined with the deviance statistic using ordinary maximum likelihood. The residuals of the model will be checked for normal distribution, which is necessary for a correctly fitted mixed model. Effect sizes will be calculated by dividing the differences between time-point and baseline estimations and the estimated baseline standard deviation. The definition of Cohen will be used for the interpretation of the effects sizes: an effect size of 0.20 is considered a small effect, 0.50 medium and 0.80 a large effect [76].

## Discussion

This paper outlines the protocol of a study evaluating the effectiveness of an intensive 1-year multidisciplinary lifestyle intervention for overweight/obese women with PCOS. Weight loss through lifestyle modification before starting fertility treatment is described as the first step for overweight/obese PCOS women who are trying to conceive [23]. However, no international evidence based protocol exists describing the design of a three-component lifestyle modification program. This is the largest RCT investigating the effectiveness of a multidisciplinary cognitive- lifestyle intervention in overweight/obese women diagnosed with PCOS. The present study started in September 2009 and the first results are expected in December 2016.

One of the strengths is the selection criterion that not only PCOS women in obesity class III, but also in obesity classes I and II, as well as overweight women are included in this study. All PCOS patients with a BMI > 25 kg/m^2^ who are eligible for ovulation induction treatment are obligated to follow the lifestyle modification protocol prior to fertility treatment. Therefore, study inclusion is not biased by weight loss motivation as in most research, as weight loss is compulsory for treatment at the Erasmus MC. This enhances the generalizability of our findings; however, this might result in higher drop-out rates.

Another strength is the length and intensity of this three-component lifestyle modification program. This 1-year program combines several evidence based elements: cognitive behavioural techniques, a multidisciplinary team, and long term support. Indeed, a recent study demonstrated that a non-invasive (two-component) 6-month weight-loss intervention preceding infertility treatment did not result in reasonable weight loss [31]. Also, the use of the oral contraceptive pill improves quality of life compared to lifestyle treatment [32]. Research supports that combining a healthy diet, increasing physical activity and behavioural modification through CBT is the best strategy for long-term weight loss [38, 41]. In addition, the SMS aims at increasing weight maintenance success and decreasing drop-out rates. As treatment adherence is often problematic [36, 42], SMS is a promising e-health tool to enhance adherence rates [50].

The goal for every participant is to achieve relevant reduction of at least 4.0 BMI points. The fertility treatment cut-off at the Erasmus MC in Rotterdam is set at 34 kg/m^2^. That means that not all women who achieve a reduction of 4.0 BMI are eligible for further fertility treatment at the Erasmus MC. We could have chosen to include this cut-off as the criterion of the primary outcome. In that case, participation would be considered a success if the weight loss was at least 4.0 BMI points AND the BMI was below 34 kg/m^2^. We chose not to include this cut-off at 34 kg/m^2^, because there is no consensus about the height of such threshold. Inclusion of this ad hoc threshold would therefore hamper the generalizability of the study. Moreover, it was considered unrealistic to expect that participants would be able to reduce more than 4.0 BMI in one year. If the participant was still above 34 kg/m^2^ after one year of treatment, the participant was encouraged to continue weight loss on their own, using the skills and attitude learned in the intervention.

Obesity increases the costs and decreases the effectiveness of fertility treatment in PCOS women [77]. The costs of this lifestyle intervention are relatively low, compared to increased medical costs in PCOS women without weight loss, as fertility treatment success is lower and the risk of pregnancy complications is higher [78].

Obesity is considered to be a multi-factorial problem; therefore, a more tailored intervention for subgroups, beyond using the BMI, is needed [79]. Recent research indicates that weight loss in the first 2 months is a good predictor for outcomes after 1 year of lifestyle intervention [80, 81]. Hence, identifying individuals at risk for being unsuccessful or those being successful, and providing tailored treatment, might be the solution for successful long-term weight loss [80]. A stepped-care model should be considered [82]. Therefore, we are planning to compare the results of the current study to other fertility clinics who have less invasive (one or two-component) weight loss consultation programs. This seems to be an ideal first step for the future development of a stepped-care multicentre RCT.

## Abbreviations

BMI: Body mass index
CBT: Cognitive behavioural therapy
COLA: CyclusOLigoAmenorroe
DEBQ: Dutch eating behaviour questionnaire
EDE-Q: Eating disorder examination questionnaire
FNAES: Fear of negative appearance evaluation scale
HRQoL: Health-related quality of life
IPAQ: International physical activity questionnaire
NEVO: Dutch food composition database
PAR-Q: Physical Activity readiness questionnaire
PCOS: Polycystic ovary syndrome
PCOSQ: Polycystic ovary syndrome questionnaire
QoL: Quality of life
RCT: Randomized controlled trial
RSES: Rosenberg self esteem scale
SF-36: Short Form 36
SMS: Short message service
WHO: World health organisation
